# Loss of NAD(H) from swollen yeast mitochondria

**DOI:** 10.1186/1471-2091-7-3

**Published:** 2006-01-24

**Authors:** Patrick C Bradshaw, Douglas R Pfeiffer

**Affiliations:** 1 Institute on Aging, University of Wisconsin-Madison, Madison, WI, USA; 2 Department of Molecular and Cellular Biochemistry, The Ohio State University, Columbus, OH, USA

## Abstract

**Background:**

The mitochondrial electron transport chain oxidizes matrix space NADH as part of the process of oxidative phosphorylation. Mitochondria contain shuttles for the transport of cytoplasmic NADH reducing equivalents into the mitochondrial matrix. Therefore for a long time it was believed that NAD(H) itself was not transported into mitochondria. However evidence has been obtained for the transport of NAD(H) into and out of plant and mammalian mitochondria. Since *Saccharomyces cerevisiae *mitochondria can directly oxidize cytoplasmic NADH, it remained questionable if mitochondrial NAD(H) transport occurs in this organism.

**Results:**

NAD(H) was lost more extensively from the matrix space of swollen than normal, condensed isolated yeast mitochondria from *Saccharomyces cerevisiae*. The loss of NAD(H) in swollen organelles caused a greatly decreased respiratory rate when ethanol or other matrix space NAD-linked substrates were oxidized. Adding NAD back to the medium, even in the presence of a membrane-impermeant NADH dehydrogenase inhibitor, restored the respiratory rate of swollen mitochondria oxidizing ethanol, suggesting that NAD is transported into the matrix space. NAD addition did not restore the decreased respiratory rate of swollen mitochondria oxidizing the combination of malate, glutamate, and pyruvate. Therefore the loss of matrix space metabolites is not entirely specific for NAD(H). However, during NAD(H) loss the mitochondrial levels of most other nucleotides were maintained. Either hypotonic swelling or colloid-osmotic swelling due to opening of the yeast mitochondrial unspecific channel (YMUC) in a mannitol medium resulted in decreased NAD-linked respiration. However, the loss of NAD(H) from the matrix space was not mediated by the YMUC, because YMUC inhibitors did not prevent decreased NAD-linked respiration during swelling and YMUC opening without swelling did not cause decreased NAD-linked respiration.

**Conclusion:**

Loss of endogenous NAD(H) from isolated yeast mitochondria is greatly stimulated by matrix space expansion. NAD(H) loss greatly limits NAD-linked respiration in swollen mitochondria without decreasing the NAD-linked respiratory rate in normal, condensed organelles. NAD addition can totally restore the decreased respiration in swollen mitochondria. In live yeast cells mitochondrial swelling has been observed prior to mitochondrial degradation and cell death. Therefore mitochondrial swelling may stimulate NAD(H) transport to regulate metabolism during these conditions.

## Background

### A difference in the mitochondrial respiratory chain of yeast and mammals

When lacking functional mitochondria the yeast *Saccharomyces cerevisiae *can grow in media containing a fermentable carbon source. Yeast can do this because of the ability to synthesize sufficient ATP by glycolysis and re-oxidize NADH reduced during glycolysis by converting pyruvate to ethanol. Yeast therefore provide a very useful genetic system to study mitochondrial bioenergetics. Electron transport in yeast mitochondria occurs, for the most part, similarly as it does in mammalian mitochondria. However, there is a striking difference. Yeast mitochondria do not contain a multi-subunit complex I NADH dehydrogenase capable of utilizing matrix space NADH to pump protons out of the matrix space as is present in mammalian mitochondria [1,2]. Instead, yeast utilize two single polypeptide NADH dehydrogenases, one facing the matrix space and the other facing the intermembrane space. Yeast NADH dehydrogenases do not pump protons, but still pass electrons from NADH to ubiquinone similarly as mammalian complex I.

### Nucleotide transporters in mitochondria

Several yeast inner mitochondrial membrane proteins able to transport nucleotides have been identified. They are all members of the mitochondrial carrier family of transport proteins. Three are isoforms of the adenine nucleotide translocator (ANT). The ANT exports ATP synthesized in the matrix space for ADP in the cytosol. A guanine nucleotide transporter brings GTP into the matrix space in exchange for GDP [3]. A mitochondrial carrier for pyrimidine nucleotides has also been identified [4]. And finally, a flavin adenine dinucleotide (FAD) transporter is responsible for maintaining a proper balance of matrix space FAD to flavin mononucleotide (FMN) [5]. Mammalian mitochondrial carriers for deoxynucleotides [6] and adenine nucleotides [7,8] have also been identified. Since mitochondria have shuttles that transport NAD(H) equivalents across the inner membrane and yeast mitochondria have NADH dehydrogenases facing both directions across the inner membrane, it is highly unlikely that rapid mitochondrial NAD(H) transport occurs during normal physiological conditions. However a slow rate of NAD transport across the mitochondrial inner membrane likely exists to replenish levels diluted by mitochondrial division. In this regard NAD transport activity has been identified in plant [9,10] and mammalian mitochondria [11].

### The yeast mitochondrial inner membrane contains an unspecific channel

The yeast mitochondrial inner membrane contains a permeability transition pore opened by ATP or respiratory substrates in the absence of phosphate [12]. This pore is alternatively called the yeast mitochondrial unspecific channel (YMUC) [13]. When mitochondria isolated from laboratory strains of yeast were suspended in an isoosmotic mannitol medium, YMUC opening caused colloid-osmotic mitochondrial swelling [12]. Mitochondria isolated from an industrial yeast strain, Yeast Foam, did not swell when the YMUC was opened in a mannitol medium [13]. However, they did swell after YMUC opening in a medium containing KCl. These results as well as other strain-dependent behaviors have caused conflicting results when characterizing yeast mitochondrial ion transport.

When yeast mitochondria were suspended in a KCl medium in the presence of the K^+ ^ionophore valinomycin, a swelling and decreased rate of ethanol respiration was observed [14]. The decreased respiration was hypothesized to be due to a loss of a component from the matrix space. The component postulated to be lost was either the ethanol dehydrogenase, Zn^2+ ^a cofactor for ethanol dehydrogenase, or NAD. The present study was performed to further investigate the mechanism of the decreased respiratory rate of yeast mitochondria oxidizing ethanol.

## Results

### A decreased rate of matrix space NAD-linked respiration occurs in the absence of phosphate

**Figure 1:**
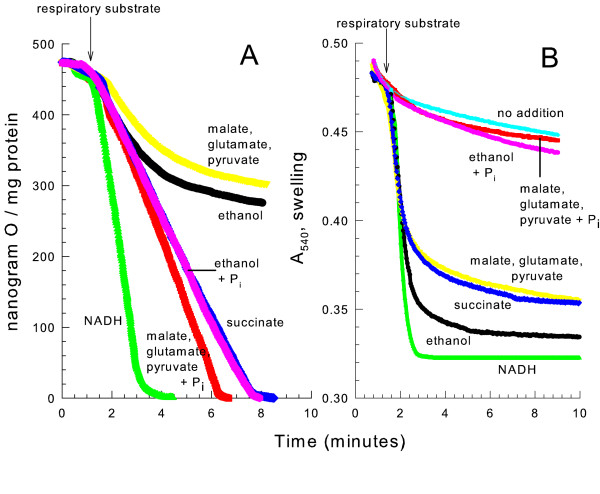
**Oxidizing NAD-linked substrates in the absence of phosphate leads to a decreased respiratory rate**. *panel A*, the medium contained 0.6 M mannitol, 10 mM HEPES (TEA^+^), pH 7.20. Where indicated 10 mM KP_i _was present. At 1.25 min. 4 mM ethanol, 0.5 mM NADH, or 5 mM succinate (K^+^), or a combination of 4 mM pyruvate (Na^+^), 4 mM malate (K^+^) and 4 mM glutamate (K^+^) were added. These concentrations were chosen to give moderate and fairly comparable rates of respiration. *panel B*, the conditions were the same as *panel A *except 32 mM ethanol was added instead of 4 mM ethanol.

When isolated yeast mitochondria were given NADH or succinate in a mannitol medium in the absence of phosphate (Fig. 1A), the respiratory (oxygen consumption) rate was nearly linear. However, when oxidizing ethanol, the respiratory rate immediately started declining, and by five minutes after ethanol addition the rate was only 10 % of the initial rate. Since external NADH and succinate were rapidly oxidized under this condition, the downstream components (ubiquinone, complex III (cytochrome reductase), cytochrome c, and complex IV (cytochrome oxidase)) of the electron transport chain maintained their functionality. Including phosphate in the medium restored a linear rate of ethanol respiration. The same pattern of respiration with ethanol was observed when yeast mitochondria oxidized the combination of malate, glutamate, and pyruvate (MGP). MGP was used as a respiratory substrate because pyruvate alone [15] or malate plus glutamate did not give a strong rate of respiration. As with ethanol, these respiratory substrates generate matrix space NADH which is oxidized by the internal NADH dehydrogenase. Therefore, NAD or the internal NADH dehydrogenase must be substantially limiting the respiratory rate when oxidizing matrix space NAD-linked substrates in the absence of phosphate.

Colloid-osmotic swelling has been shown to occur due to opening of the YMUC when oxidizing ethanol in the absence of phosphate. Therefore, light scattering of the mitochondrial suspension was monitored under identical conditions to follow mitochondrial volume changes (Fig. 1B). Oxidizing any of these respiratory substrates caused mitochondrial swelling. The rate of swelling was slightly greater for mitochondria oxidizing the given concentrations of NADH or ethanol as compared to the given concentrations of succinate or MGP. This swelling was blocked by phosphate. Therefore the decreased NAD-linked respiratory rate is either due to YMUC opening or mitochondrial swelling.

### The decreased respiratory rate is independent of YMUC opening

**Figure 2:**
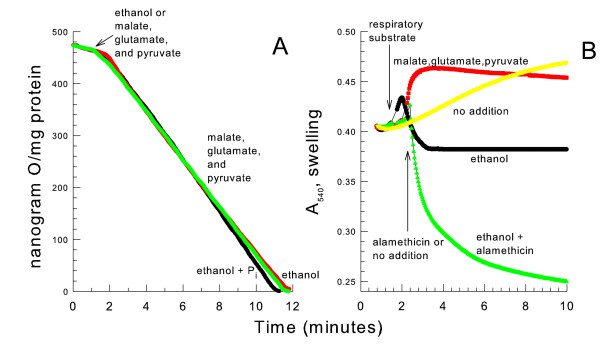
**The decreased respiratory rate does not occur when oxidizing NAD-linked substrates in a sucrose medium**. *panel A*, the medium contained 0.6 M sucrose, 10 mM HEPES (TEA^+^), pH 7.20. Where indicated 10 mM KP_i _was present. 4 mM ethanol or a combination of 4 mM pyruvate (Na^+^), 4 mM malate (K^+^) and 4 mM glutamate (K^+^) were added where indicated. *panel B*, the medium was the same as *panel A *except 32 mM ethanol was added instead of 4 mM ethanol.

In contrast to results obtained in a mannitol medium, a linear rate of respiration occurred when mitochondria were given ethanol or MGP and were suspended in a sucrose medium in the absence of the YMUC inhibitor phosphate (Fig. 2A). Mitochondrial swelling did not occur when the YMUC was opened in the sucrose (342 Da) medium unless the pore-forming antibiotic alamethicin was present (Fig. 2B). In fact an increase in light scattering indicating a mitochondrial contraction often occurred. A decreased mitochondrial volume may occur if sucrose is unable to permeate through the YMUC and endogenous potassium salts exit the matrix space upon YMUC opening. This data suggests that YMUC opening is not responsible for the greatly decreased rate of respiration.

In the presence of phosphate, the mitochondria had a slower linear rate of respiration on NAD-linked substrates in the sucrose medium than in the mannitol medium (compare Fig. 1A to Fig. 2A). This may be related to the decreased initial absorbance (increased apparent initial matrix space volume) when mitochondria were suspended in the sucrose medium. However after spontaneously contracting for 10 minutes a volume similar to the initial absorbance of mitochondria suspended in a mannitol medium was obtained.

Mg^2+ ^(size ~24 Daltons) was not extensively released from the mitochondrial matrix after ethanol-induced YMUC opening like it was after ATP-induced YMUC opening (see additional file 1: Figure S1). Therefore the respiration-induced YMUC does not transport solutes as large as previously reported (~1,000 Daltons) [12].

### Mitochondrial swelling without YMUC opening causes decreased respiration

**Figure 3:**
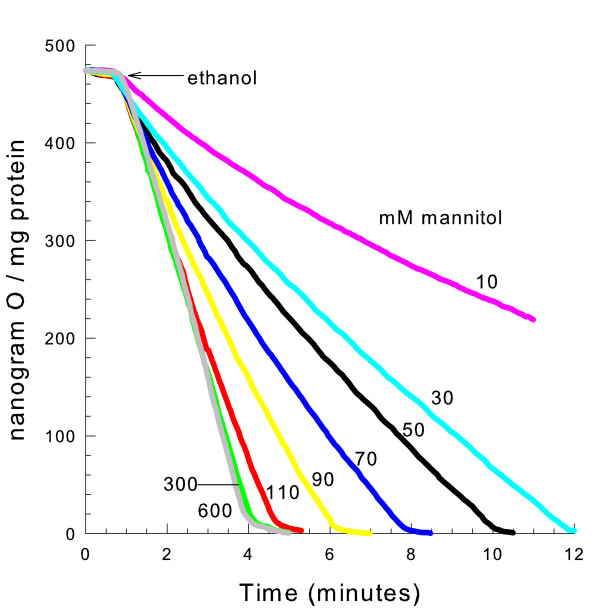
**Hypotonic swelling induces a decreased rate of ethanol respiration**. The medium contained 10 mM HEPES (TEA^+^), pH 7.20, and 0.1 mM decavanadate (Na^+^). Where indicated 10, 30, 50, 70, 90, 110, 300, or 600 mM mannitol was present. 32 mM ethanol was added as shown.

Since YMUC opening does not necessarily lead to decreased ethanol respiration, we sought to determine if mitochondrial swelling was sufficient to induce the decreased respiratory rate. To obtain an incremental amount of mitochondrial swelling, mitochondria were suspended in mannitol media of decreasing osmotic strength. The lower the osmotic strength the greater the volume of the matrix space of isolated mitochondria in suspension. Decavanadate, a potent YMUC inhibitor [16], was present at a concentration to completely prevent opening of the YMUC (see additional file 1: Figure S2). As shown in Fig. 3, mannitol concentrations less than 110 mM yield a concentration-dependent decrease in the rate of respiration. Therefore swelling, not YMUC opening is responsible for the decreased respiratory rate.

### NAD addition restores the respiratory rate of swollen mitochondria

**Figure 4:**
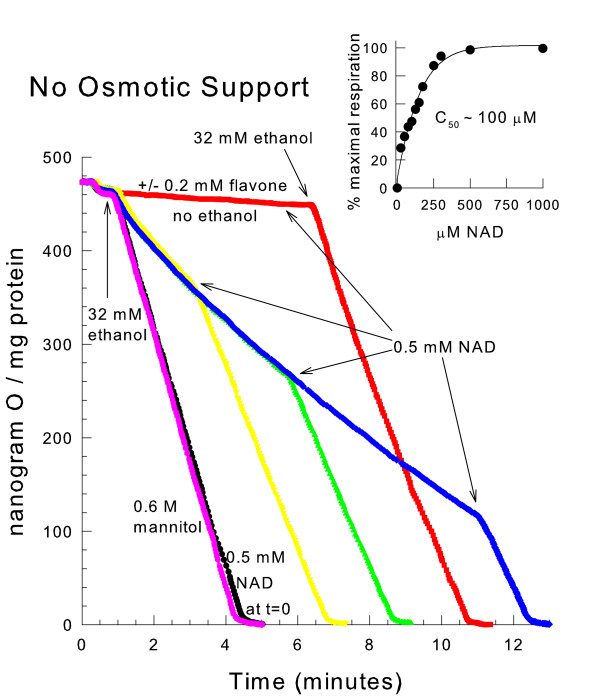
**NAD addition restores the respiratory rate of hypotonically swollen mitochondria**. The medium contained 10 mM mannitol, 10 mM HEPES (TEA^+^), pH 7.20 and 0.1 mM decavanadate (Na^+^). 0.6 M mannitol was present where indicated. 0.5 mM NAD was added where indicated by an arrow or present from the beginning where indicated by t = 0. 32 mM ethanol was added at either 45 seconds or 7 minutes as indicated. *inset*, the conditions were the same as in the figure. The NAD concentration present was varied. The extents of oxygen consumption at 4 min. were compared.

To determine if NAD was limiting the respiratory rate under conditions of mitochondrial swelling, NAD was added to mitochondria that were osmotically swollen in a hypotonic medium in the presence of the YMUC inhibitor decavanadate. As shown in Fig. 4*inset*, the addition of NAD completely restored the rate of ethanol respiration in a concentration dependent manner. Since NAD was also able to restore the respiratory rate in the presence of 0.2 mM flavone, a membrane impermeant NADH dehydrogenase inhibitor [17,18], the added NAD appears to be transported into the matrix space. Once there, the NAD is reduced and donates electrons to the internal NADH dehydrogenase. The addition of NAD was able to restore the respiratory rate when added at any time after ethanol addition (Fig. 4). However, NAD was not able to restore the respiratory rate of swollen mitochondria oxidizing MGP (data not shown). Therefore, other factors necessary for the activity of matrix space dehydrogenases such as Krebs cycle metabolites or enzyme cofactors may be released from the mitochondria during swelling as well.

**Figure 5:**
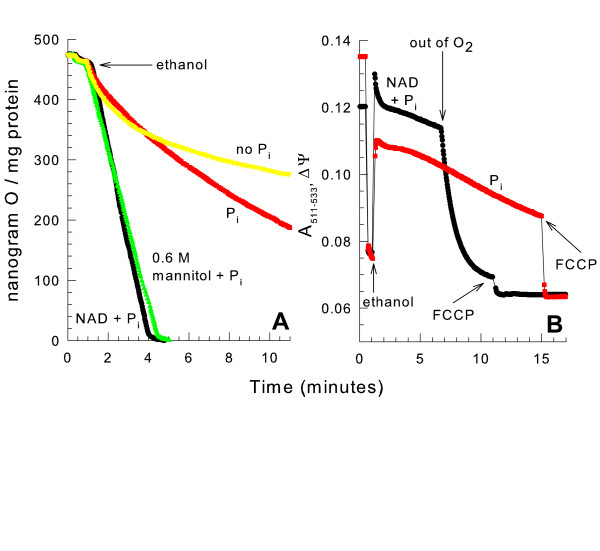
**A membrane potential is maintained in the presence of decreased respiration**. *panel A*, the medium contained 10 mM mannitol, 10 mM HEPES (TEA^+^), pH 7.20. Where indicated 0.6 M mannitol, 10 mM KP_i _or 500 μM NAD was present. 32 mM ethanol was added as shown. *panel B*, the conditions were the same as *panel A *except the membrane potential was monitored, 12 μM safranine O was present, and 4 μM FCCP was added where indicated.

We next monitored the mitochondrial membrane potential during the decreased respiratory rate of yeast mitochondria oxidizing ethanol suspended in a hypotonic medium. It has been shown that yeast mitochondria need greater than 1 mM phosphate to prevent YMUC opening and membrane potential dissipation when oxidizing ethanol [12]. Therefore, we monitored the respiratory rate and membrane potential in the presence of 10 mM phosphate, a quantity sufficient to keep the YMUC completely closed [12]. The hypotonically swollen mitochondria showed a decreased rate of ethanol respiration in the presence of phosphate that could be restored by NAD addition to the medium (Fig. 5A). The membrane potential in these hypotonically swollen mitochondria was also monitored (Fig. 5B). Even though the respiratory rate was much slower in swollen mitochondria without added NAD, a membrane potential that slowly decreased over time was still present. The membrane potential of the swollen mitochondria in the presence of NAD was almost identical to that generated by normal, condensed mitochondria suspended in a mannitol medium (data not shown). The uncoupler FCCP (carbonylcyanide-p-trifluoromethoxyphenylhydrazone) was able to fully dissipate the membrane potential. Therefore, during the decreased respiratory rate the YMUC was not open and protons were not freely permeable to cross the inner membrane. The majority of swollen yeast mitochondria remain physically intact following swelling because they are able to maintain large amplitude contraction after colloid-osmotic [12] or hypotonic [19] swelling. The ability of NAD to completely restore the respiratory rate in swollen mitochondria is also an indication of mitochondrial inner membrane integrity.

### Mitochondrial contraction after swelling does not restore the respiratory rate

**Figure 6:**
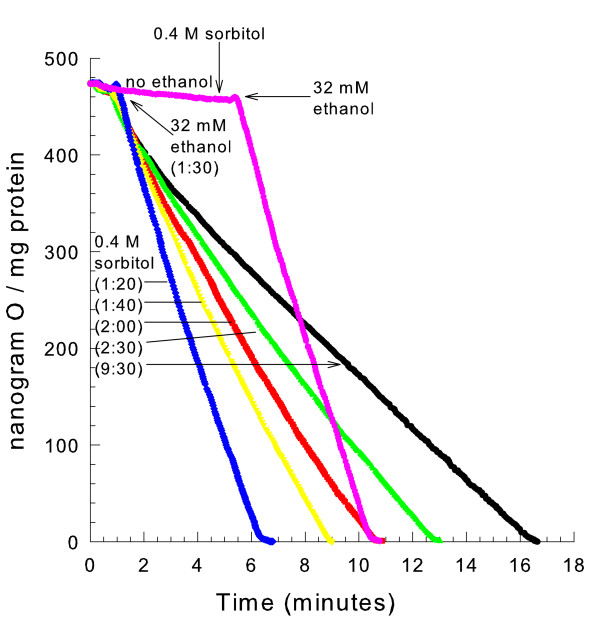
**Osmotic support can not fully restore the decreased respiratory rate in mitochondria oxidizing ethanol when swollen**. The medium contained 10 mM mannitol, 10 mM HEPES (TEA^+^), pH 7.20 and 0.1 mM decavanadate (Na^+^). 32 mM ethanol was added at 1:30 or 5:30 as indicated. 0.4 M sorbitol was added at either 1:20, 1:40, 2:00, 2:30, 9:30, or 5:00 as indicated.

When mitochondria swell, the volume of the matrix space expands, diluting the contents of the matrix space. It could be possible that NAD dilution limits the rate of ethanol respiration. To test this hypothesis mitochondria were osmotically swollen in a hypotonic medium, given a respiratory substrate, and then contracted by the addition of 0.4 M sorbitol. If dilution of matrix space contents caused the decreased respiratory rate, then subsequent contraction should restore the rate. As shown in Fig. 6, adding osmotic support 8 minutes after ethanol addition did not restore the rate of respiration. However, addition of sorbitol at 10, 30, or 60 seconds after ethanol addition partially restored the respiratory rate in a time-dependent manner. This is not consistent with a dilution of metabolites in the expanded matrix space because mitochondrial volume changes occur nearly instantaneously with the osmotic changes in the medium.

Hypotonically swelling mitochondria for 4.5 minutes and then recontracting them by the addition of osmotic support (sorbitol) before ethanol addition did not result in a decreased respiratory rate once ethanol was added (Fig. 6). Therefore, not only is swelling needed for the decreased respiratory rate, but also respiration must be occurring during the time of swelling for the decreased respiratory rate to occur. So the loss of NAD from the matrix space of swollen mitochondria appears to be a respiration-dependent process.

### NAD(H) is released from swollen mitochondria

**Figure 7:**
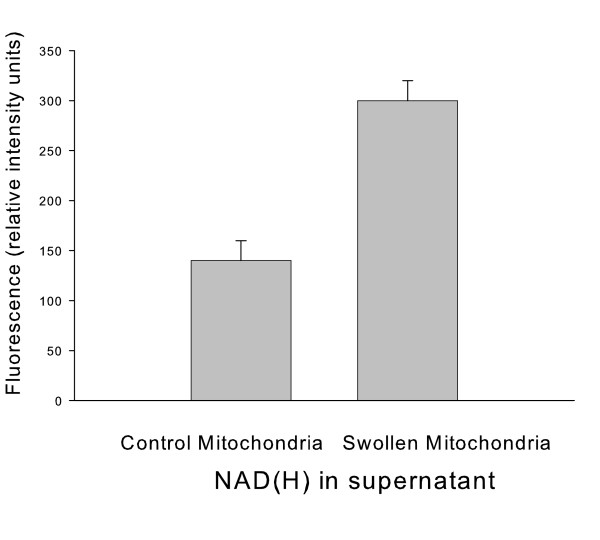
**Swelling stimulates NAD(H) release from mitochondria**. Mitochondria were suspended in 0.6 M mannitol, 10 mM HEPES (TEA^+^), pH 7.20. 2 mM ethanol was added to the mitochondria in either the absence or presence of 10 mM KP_i _to allow or not allow swelling respectively. The mitochondria were then spun down. The supernatant was extracted, the NAD reduced, and the fluorescence assayed.

Since mitochondrial NAD(H) could be either degraded or released from the matrix space, extramitochondrial NAD(H) levels were measured. For these experiments, mitochondria were either swollen by the addition of ethanol or maintained unswollen by the addition of both ethanol and 10 mM phosphate [12]. The combination of extramitochondrial NAD plus NADH was measured by fluorescence spectroscopy from the mitochondrial supernatant after spinning mitochondria out of the medium and reducing NAD. As shown in Fig. 7, the NAD(H) level in the supernatant obtained from the suspension of swollen mitochondria was twice the amount compared to that obtained from unswollen mitochondria. The NAD(H) in the supernatant from unswollen mitochondria was likely due to a slower leak from the matrix space in these organelles. But not enough NAD(H) release occurred in these condensed mitochondria to decrease NAD-linked respiration.

**Figure 8:**
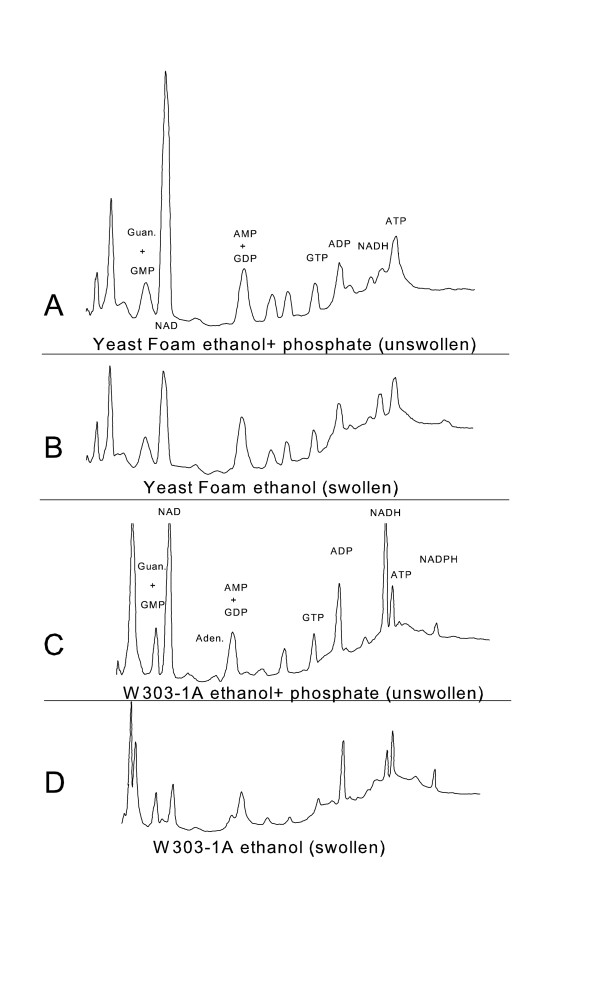
**Nucleotide profile of swollen and unswollen mitochondria**. The medium contained 0.6 M mannitol, 10 mM HEPES (TEA^+^), pH 7.20, and 15 μg/ml oligomycin. 2 mM ethanol was added to the mitochondria in the absence or presence of 10 mM KP_i _and incubated for 10 minutes. Mitochondria were then extracted for HPLC analysis. Mitochondria isolated from strains W303-1A or Yeast Foam were used as indicated. A numerical comparison of the nucleotide release from the swollen and unswollen mitochondria is shown in additional file 1: Table S1. Results are representative of 3 experiments from W303-1A and 2 experiments from Yeast Foam. Trends were conserved in the different experiments whereas exact values showed moderate variation.

To address the question as to the amount of NAD(H) remaining in the mitochondrial matrix space after swelling, and to determine if other nucleotides are released during swelling, total nucleotides in the mitochondrial pellet were measured by reversed phase high performance liquid chromatography (HPLC) in swollen and normal, condensed mitochondria. Mitochondrial nucleotides from both the W303-1A strain and the Yeast Foam strain were measured. As shown in Fig. 8 the majority of nucleotides remained at comparable levels whether mitochondria were swollen by ethanol addition or kept in the normal, condensed state by the presence of ethanol and phosphate. GTP and ADP were present at a modestly reduced level in swollen mitochondria from the W303-1A strain, while there was variability in the NADH response to swelling between yeast strains. However in both strains, the most striking difference between swollen and unswollen conditions was that the NAD peak was only about one third as large in swollen mitochondria as compared to unswollen mitochondria. Therefore swelling results in decreased levels of NAD(H) in the mitochondrial matrix.

## Discussion

A decreased rate of ethanol respiration occurs when mitochondria are suspended in a KCl medium in the presence of valinomycin presumably due to a loss of matrix space components [14]. Our data indicate that NAD(H) is the component lost and it is lost from swollen mitochondria independently of the opening of the YMUC. Since NAD addition can restore the respiratory rate in the presence of a membrane impermeant NADH dehydrogenase inhibitor, external NAD appears to enter the matrix space in swollen mitochondria. The metabolite loss is not completely specific for NAD since NAD addition does not restore MGP respiration in swollen mitochondria. But the loss has some degree of specificity because many nucleotide species are not lost upon mitochondrial swelling (see Fig. 8).

### Significance of a mitochondrial NAD(H) transport activity

We believe that a transport of NAD(H) across the inner mitochondrial membrane is the most probable explanation for our data, although other interpretations are possible. A mitochondrial NAD transport activity may be needed to restore matrix space NAD levels diluted by mitochondrial division. Our data indicate that the swelling-induced NAD transport activity may be minimally active in normal, condensed mitochondria to fulfill this role, because a substantial amount of NAD(H) is released from normal, condensed mitochondria, even if NAD-linked respiration is not decreased in these unswollen organelles.

Stimulation of an NAD(H) transport activity in the inner membrane upon mitochondrial swelling would be of physiological relevance since the equilibration of NAD(H) between mitochondria and the cytoplasm may allosterically inhibit or activate certain enzymes. In this regard the SIRT family of NAD-dependent histone deacetylases regulates aging, the cell cycle, and apoptosis [20]. SIR2, a yeast homolog controls the replicative lifespan of yeast. Therefore the cytoplasmic level of NAD(H) may be very important to many aspects of cell growth.

The equilibration of NAD(H) between the cytoplasm and the mitochondrion may also be important in coordinating energy metabolism with nutrient availability. Under conditions of glucose excess for example, the YMUC may open because the cytoplasmic phosphate concentration drops from 4 mM to 1–2 mM [21,22] causing mitochondrial swelling [23]. The majority of cellular NAD(H) resides in mitochondria [24]. Therefore, an equilibration of NAD(H) between compartments would likely increase the cytoplasmic concentration. This loss of mitochondrial NAD(H) and other tricarboxylic acid cycle intermediates or cofactors would slow the tricarboxylic acid cycle so NAD would be reduced at a slower rate. This would be beneficial to slow respiration and conserve energy when the YMUC is open and dissipating the proton gradient.

The addition of glucose to yeast growing on a nonfermentable carbon source results in the swelling of a portion of the mitochondria [23]. During this change in carbon source the cell switches from a mostly aerobic metabolism to a highly glycolytic metabolism. It has been estimated that during this change in metabolism mitochondria decrease from 12 % of the cell volume to 3% of the cell volume [25]. A switch to a fermentable substrate is also accompanied by a release of lytic enzymes from the vacuole [23] followed by a release of mitochondrial proteins into the cytoplasm, which are then either degraded or imported back into remaining mitochondria [26]. An increase in cytoplasmic NAD due to its mitochondrial release may stimulate glycolysis or act as a cytoplasmic signaling molecule during such conditions.

The structure of yeast mitochondria during swelling and subsequent contraction has been studied using electron tomography [27]. After swelling and contraction most of the mitochondrial cristae reform, but contain no openings to the intermembrane space. Therefore swelling may cause irreversible structural damage to mitochondria resulting in cytoplasmic ATP depletion and cell death.

It is possible that the irreversible change in membrane structure during mitochondrial swelling may also play a role in the stimulation of NAD(H) transport activity.

### Yeast mitochondrial swelling during programmed cell death

Yeast mitochondria have been shown to swell during programmed cell death [28]. Mammalian mitochondria swell under conditions that induce necrotic cell death as a result of permeability transition pore opening [29]. Since cytochrome c is dispensable for cell death induced by expression of the proapoptotic Bax protein in yeast, it is uncertain as to what function mitochondrial swelling would serve in yeast cell death [30]. It is possible that other yeast proteins from the mitochondrial intermembrane space are involved in this process. In either case, NAD(H) may be released from the matrix space and alter cellular metabolism to influence yeast cell death.

### Solute selectivity of the respiration-induced YMUC

It has previously been reported that the respiration-induced YMUC is able to transport solutes up to 1000 Daltons using a solute exclusion method with different molecular weights of polyethylene glycol (PEG) [12]. However there is also evidence that PEG molecules of molecular weight less than 1000 Daltons are membrane permeable [31], which would question the suitability of this method for the size determination of small pores. Consistent with this previous observation, yeast mitochondria spontaneously swell when they are suspended in a medium containing 400 or 600 molecular weight PEG indicating that these PEG molecules may be membrane permeant. YMUC opening by ethanol addition does not increase the rate of swelling (see additional file 1: Figure S3) suggesting PEG-400 and PEG-600 are too large to be transported by YMUC.

Mitochondrial nucleotides have also been shown to be released from the YMUC [12]. However, repeating these mitochondrial nucleotide level measurements using a shorter incubation period this time (10 minutes) revealed no large-scale efflux of nucleotides. It is possible that mitochondrial phospholipid hydrolysis from the large yeast mitochondrial phospholipase A activity [32] and long incubation time previously employed led to a loss of inner membrane integrity and nucleotide efflux. Since Mg^2+^, sucrose, PEG-400, PEG-600 and other small molecules (EDTA and raffinose, data not shown) are not permeable through the respiration-induced YMUC, nucleotides that are of a slightly larger size are almost certainly not permeable. Therefore the nucleotides that are lost from the mitochondria during YMUC opening and swelling appear to be lost independently of YMUC opening. The ability to maintain a linear rate of NAD-linked respiration after YMUC opening in a sucrose medium is further evidence that YMUC opening by itself does not allow loss of NAD from the matrix space.

The Ndt1 (*YIL006W*) and Ndt2 (*YEL006W*) genes have recently been identified as encoding yeast mitochondrial NAD transporters [33]. Both proteins are excellent candidates to mediate a mitochondrial swelling stimulated transport of NAD. Reconstituted Ndt1 transported NAD rapidly when in exchange for another nucleotide or slowly by a uniport mechanism. It was suggested that NAD transport into the matrix space in yeast cells occurs most frequently in exchange for AMP or GMP. The Ndt1 Ndt2 double mutant had a slow growth phenotype when growing on nonfermentable carbon sources but still maintained a low level of mitochondrial NAD. This suggests that another transporter aids Ndt1 and Ndt2 in maintaining basal matrix space NAD levels.

## Conclusion

NAD(H) loss from isolated yeast mitochondria is stimulated upon times of matrix space expansion. Added NAD is able to restore the decreased respiratory rate of swollen mitochondria by entering the matrix space, being reduced and donating electrons to the internal NADH dehydrogenase. Therefore NAD appears to be transported across the inner membrane in swollen organelles. Since yeast mitochondria contain NADH dehydrogenases facing both the matrix space and intermembrane space, and the matrix space is normally in a condensed state, a mitochondrial transport of NAD(H) is only likely to occur at a minimal rate under normal conditions. This may be enough to maintain matrix space NAD(H) levels throughout mitochondrial divisions. NAD(H) transport activity would also be stimulated when mitochondria swell prior to their degradation. Mitochondrial swelling is known to occur during switches from nonfermentable to fermentable carbon sources as well as prior to yeast cell death. The redistribution of cellular NAD(H) during these times may facilitate the metabolic changes needed for these processes to occur.

## Methods

### Yeast growth and mitochondrial isolation

The yeast strains W303-1A and Yeast Foam were grown aerobically at 30°C in a medium containing 2% lactate, 1% yeast extract, 2% peptone, 0.05% dextrose, and 0.01% adenine at pH 5.0. Yeast cells were harvested during logarithmic growth phase (A_600 _= 1.8–2.2). Mitochondria were isolated from spheroplasts as previously described [34], except 0.6 M sucrose was used in the homogenization buffer instead of 0.6 M mannitol. The isolated yeast mitochondria were suspended in 0.6 M mannitol, 20 mM HEPES (TEA^+^), and 0.1 mM EGTA, pH 6.8. Protein concentration was determined by a mini-biuret method using BSA as the standard. Briefly, 25 μl of mitochondrial suspension was added together with 75 μl of 10 % Na^+ ^deoxycholate. Then 900 μl of biuret reagent was added. The mixture was incubated for 15 minutes, and the absorbence read at 540 nm.

### Mitochondrial light scattering, respiration, and membrane potential determination

Solute permeability was monitored by light scattering using an SLM-Aminco DW-2C spectrophotometer in split beam mode at A_540 _using mitochondria suspended at 1 mg protein /ml. Respiration was monitored with a Clark oxygen electrode (Yellow Springs) suspending the mitochondria at a concentration of 1 mg/ml at 25°C. The mitochondrial membrane potential was monitored by absorbance of the dye safranine O as described in [35].

### Decavanadate solution

A decavanadate solution was made from a 0.1 M stock solution of Na^+ ^orthovanadate adjusted to pH 6.0 to obtain an orange color typical of this polymeric species and then further diluted [16].

### Determination of nucleotides

Total NAD plus NADH fluorescence measurements were performed as described in [36]. High performance liquid chromatography analysis of the yeast mitochondrial nucleotides was performed after an alkaline extraction of the mitochondrial suspension, using a C_18 _reversed phase column on a Beckman model 110A instrument. Nucleotide retention times were monitored using a UV detector at A_280 _[12].

## Authors' contributions

PB designed and performed the experiments and wrote the manuscript. DP supervised the work.

## Supplementary Material

Additional File 1 Figure S1 ATP-induced opening but not ethanol-induced opening of YMUC stimulates mitochondrial Mg^2+ ^efflux. Figure S2 Decavanadate inhibits the respiration-induced YMUC. Figure S3 0.4 and 0.6 kD PEG are not permeable through the respiration-induced YMUC. Table S1 Nucleotide levels in swollen mitochondria. Click here for file
