# Supplementary material for: Loss of NAD(H) from swollen yeast mitochondria
Source: BMC Biochem. 2006 Jan 24;7:3. doi: 10.1186/1471-2091-7-3 (PMC1395316; doi:10.1186/1471-2091-7-3)
Supplement: Additional File 1 — Figure S1 ATP-induced opening but not ethanol-induced opening of YMUC stimulates mitochondrial Mg2+ efflux. Figure S2 Decavanadate inhibits the respiration-induced YMUC. Figure S3 0.4 and 0.6 kD PEG are not permeable through the respiration-induced YMUC. Table S1 Nucleotide levels in swollen mitochondria. [file 1471-2091-7-3-S1.pdf]

Additional file 1

for

Loss of NAD(H) from Swollen Yeast Mitochondria

By Patrick C. Bradshaw and Douglas R. Pfeiffer

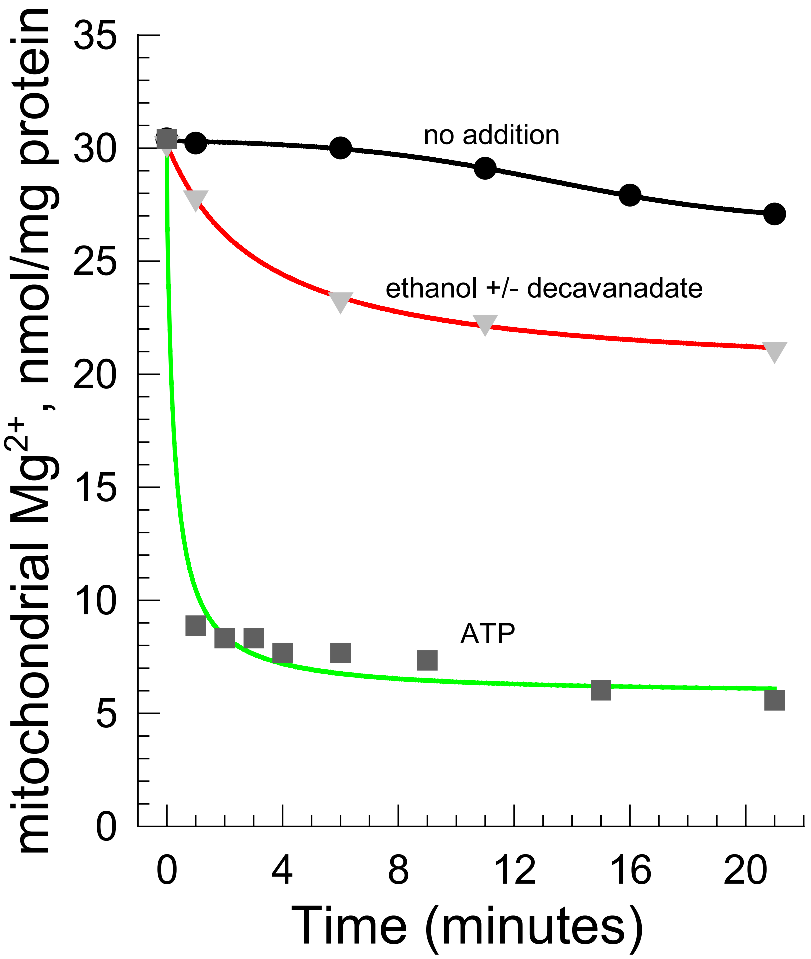

Figure S1 ATP-induced opening but not ethanol-induced opening of YMUC stimulates mitochondrial Mg^{2+} efflux. The medium contained 0.6 M mannitol, 10 mM HEPES (TEA^+), pH 7.20. 0.1 mM decavanadate (Na^+) was present where shown. Where indicated 1 mM ethanol or 2 mM ATP (Na^+) were present. For ion concentration determination atomic absorption spectrophotometry was performed using an AA-575 spectrophotometer (Varian). The mitochondrial suspension was spun down for 2 minutes in a microcentrifuge and the supernatant poured off. The mitochondrial pellet was solubilized with 0.5 mL 2 N perchlorate overnight and then diluted with deionized water up to 2 mL for sample reading.

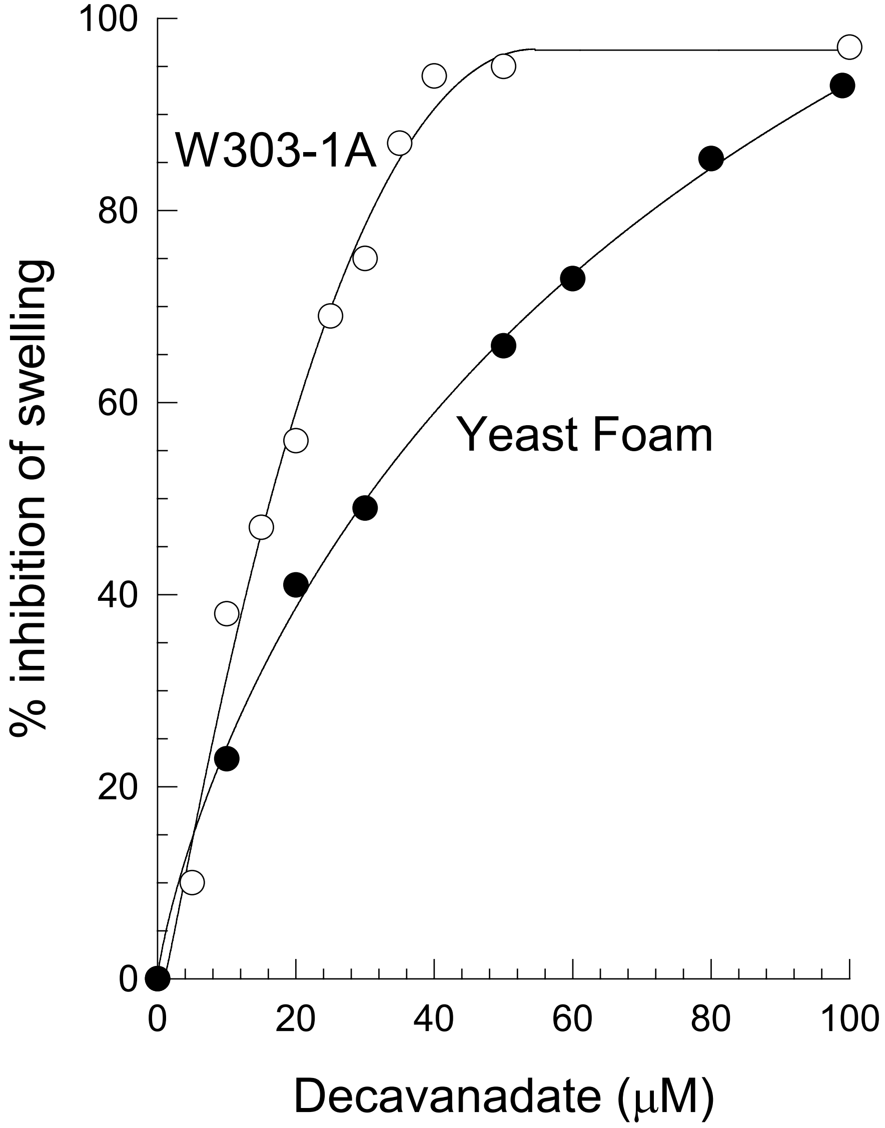

Figure S2 Decavanadate inhibits the respiration-induced YMUC. The medium contained 0.6 M mannitol, 10 mM HEPES (TEA^+), pH 7.20, 1 mM ethanol, 15 μg/ml oligomycin, and the amount of decavanadate (Na^+) indicated.

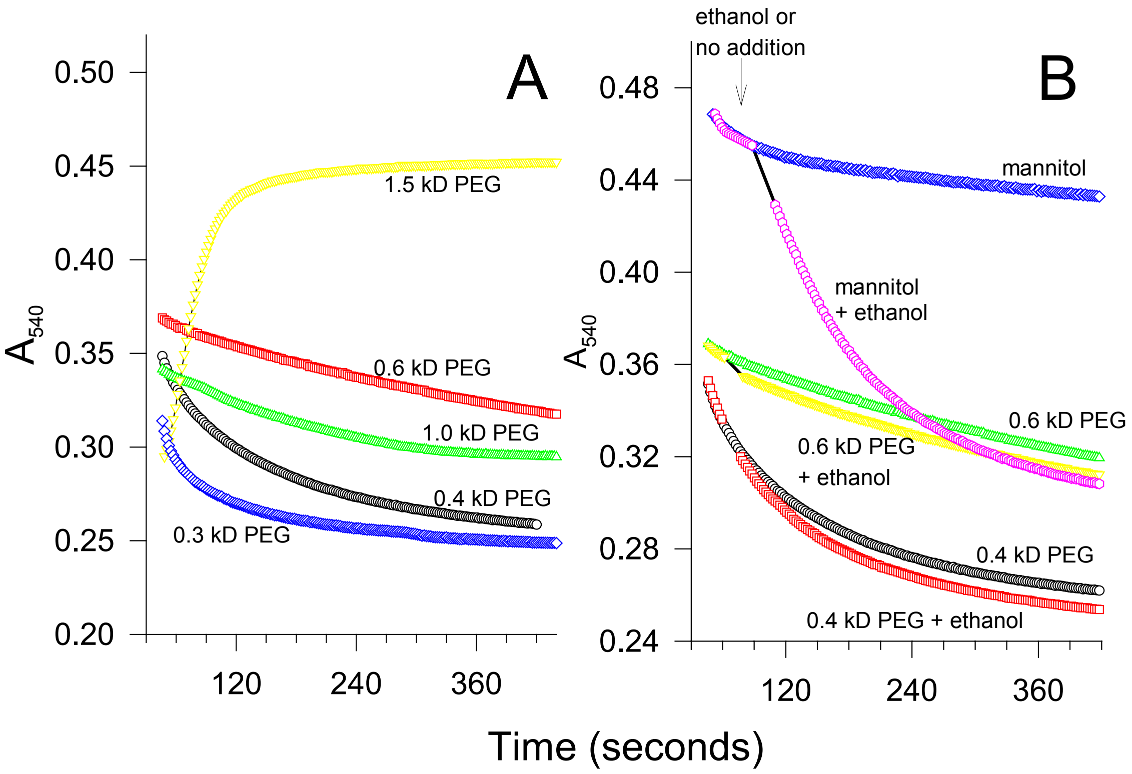

Figure S3 0.4 and 0.6 kD PEG are not permeable through the respiration-induced YMUC. *panel A*, the medium contained either 0.4 osM 0.3, 0.4, 0.6, 1.0 or 1.5 kD PEG, 5 mM HEPES (TEA^+), and 0.1 mM decavanadate (Na^+). *panel B*, the mitochondria were suspended in 0.4 osM 0.4 or 0.6 kD PEG, 5 mM HEPES (TEA^+). 1 mM ethanol or no addition was made at 90 seconds.

Nucleotide	Percent of level in unswollen mitochondria	
	W303-1A	Yeast Foam
Guanosine + GMP	60	82
NAD	22	36
AMP + GDP	84	101
GTP	34	72
ADP	57	91
NADH	26	340
ATP	83	70
NADPH	82	107

Table S1 Nucleotide levels in swollen mitochondria. The peak areas from Figure 8 were determined using Origin graphing software (OriginLab).
